# Supplementary material for: An ethologically relevant paradigm to assess defensive response to looming visual contrast stimuli
Source: Sci Rep. 2024 May 31;14:12499. doi: 10.1038/s41598-024-63458-1 (PMC11143276; doi:10.1038/s41598-024-63458-1)
Supplement: Supplementary file 1 — Supplementary Information. [file 41598_2024_63458_MOESM1_ESM.pdf]

Response frequency and C50 for individual animals

A

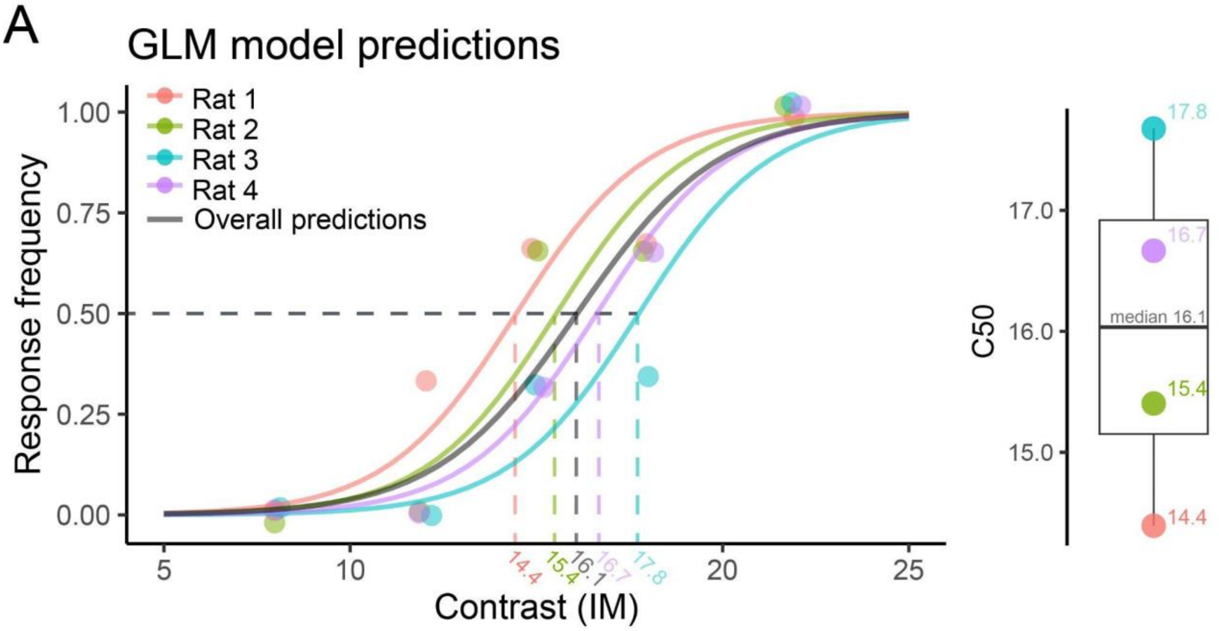

B

LTCV results for Rat 1

Repetition	Contrast (MI)				
	8	12	15	18	22
1°	No response	No response	Head Bobbing	Rearing	Freezing
2°	No response	No response	No response	Head Bobbing	Freezing
3°	No response	Rearing	Rearing	No response	Rearing
Total responses	0	1	2	2	3
Frequency	0.00	0.33	0.67	0.67	1.00

Supplementary Figure 1. A) Individual GLM model predictions and C50 values for 4 randomly chosen rats. B) Raw data and Frequency for rat 1, sorted by contrast level.

Luminosity of experimental arenas and Contrast calculations

Experimental arenas luminance and contrasts indexes							
Mice arena				Rats arena			
Contrast level	Luminosity (Lux)	Michelson Index (%)	Weber contrast	Contrast level	Luminosity (Lux)	Michelson Index (%)	Weber contrast
Background	39.4	0	0	Background	39.4	0	0
1	36.4	3.96	0.08	1	33.6	7.95	0.17
2	34.9	6.06	0.13	2	30.9	12.09	0.28
3	33.6	7.95	0.17	3	29.1	15.04	0.35
4	32.2	10.06	0.22	4	27.4	17.96	0.44
5	30.9	12.09	0.28	5	25.2	21.98	0.56
6	28.5	16.05	0.38				

Supplementary table 1: Luminosity was measured with a photometer placed in the center of the arena at the floor level. Luminosity is shown for the background (the light in the arena when no stimulus is being displayed), and for each contrast level when the disk is fully expanded. Also, two indices for contrast were calculated. Michelson index (%) calculated as (Background-Disk)*100/(Disk+Background), and Weber contrast, calculated as (Background - Disk)/Disk.

Model selection and contrasts for naive rats experiments (Figure 3)

Model selection on Naive rats experiments									
Bottom up construction of models			anova between models (Test: Chisq)						
	Fixed effects	AIC	Model	Resid df.	Resid. Dev	df	Deviance	Pr(>Chi)
Model 1	Contrast	192	Model 1	128	110.14	1		
Model 2	Contrast + Sex	189	Model 2	127	104.92	1	5.22	0.022
Model 3	Contrast + Sex + Age	168	Model 3	126	85.56	1	22.37	<0.0001
Model 4	Contrast + Sex + Age + Time of the day	162	Model 4	125	74.11	1	8.43	0.0037

Supplementary table 2: Model selection for experiments on naive rats. Model 4 was chosen as the best model.

Contrasts between factors for the best model							
Fixed effect		Prob	Std. Error	LCI	UCI	Odds Ratio	p
Sex	Female	0.65	0.11	0.42	0.83	2.32	0.043
	Male	0.46	0.06	0.33	0.56		
Age	Young	0.72	0.05	0.62	0.80	4.35	0.0026
	Old	0.37	0.12	0.17	0.62		
Time of the day	16 ZT	0.70	0.11	0.46	0.86	3.66	0.005
	6 ZT	0.39	0.06	0.29	0.50		

Supplementary table 3: Contrasts performed for model 4. LCI and UCI: Lower and upper 95% confidence intervals

Model selection and contrasts for ischemic rats experiments (Figure 4)

Model selection on ischemia experiments										
Bottom up construction of models				anova between models (Test: Chisq)						
	Fixed effects	AIC		Model	Resid df.	Resid. Dev	df	Deviance	Pr(>Chi)
Model 1	Contrast	147		Model 1	83	84.91			
Model 2	Contrast + Treatment	115		Model 2	81	48.69	2	36.22	<0.0001

Supplementary table 4: Model selection for experiments on ischemic rats. Model 2 was chosen as the best model.

Contrasts between factors for the best model							
Fixed effect		Prob	Std. Error	LCI	UCI	Odds Ratio	p
Treatment	C	0.44	0.07	0.31	0.58	15.28 (C vs Bilat)	<0.0001
	Unilat	0.2	0.05	0.11	0.34	3.04 (C vs Unilat)	0.034
	Bilat	0.05	0.02	0.02	0.11	0.20 (Unilat vs Bilat)	0.0036

Supplementary table 5: Contrasts performed for model 2. C: Control, Unilat: Unilateral Ischemia, Bilat: Bilateral Ischemia. LCI and UCI: Lower and upper 95% confidence intervals

Model selection and contrasts for naive mice experiments (Figure 5)

Model selection on Naive mice experiments									
Bottom up construction of models				anova between models (Test: Chisq)					
	Variables	AIC		Model	Resid df.	Resid. Dev	df	Deviance	Pr(>Chi)
Model 1	Contrast	367		Model 1	166	200.42			
Model 2	Contrast + Sex	312		Model 2	165	192.97	1	7.45	0.006
Model 3	Contrast + Sex + Age	308		Model 3	164	137.34	1	55.63	<0.0001

Supplementary table 6: Model selection for experiments on naive mice. Model 3 was chosen as the best model.

Contrasts between factors for the best model							
Fixed effect		Prob	Std. Error	LCI	UCI	Odds Ratio	p
Sex	Female	0.52	0.03	0.45	0.58	1.62	0.0211
	Male	0.40	0.04	0.36	0.47		
Age	Young	0.65	0.03	0.58	0.71	4.79	<0.0001
	Old	0.28	0.03	0.21	0.34		

Supplementary table 7: Contrasts performed for model 2. LCI and UCI: Lower and upper 95% confidence intervals

Model selection and contrasts for tg mice experiments (Figure 6)

Model selection on Tg mice experiments									
Bottom up construction of models				anova between models (Test: Chisq)					
	Variables	AIC		Model	Resid df.	Resid. Dev	df	Deviance	Pr(>Chi)
Model 1	Contrast	548		Model 1	208	467.62			
Model 2	Contrast + Genotype	262		Model 2	202	169.44	6	298.2	<0.0001

Supplementary table 8: Model selection for experiments on tg mice. Model 2 was chosen as the best model.

Contrasts between factors for the best model					
Fixed effect		Prob	Std. Error	LCI	UCI
Genotype	WT	0.67	0.04	0.54	0.78
	RKO	0.66	0.06	0.45	0.81
	C only	0.63	0.04	0.51	0.73
	CKO	0.15	0.08	0.08	0.26
	R only	0.09	0.07	0.03	0.23
	M only	0.01	0.02	0.01	0.03
	TKO	0	-	-	-

Supplementary table9: Contrasts performed for model 2. LCI and UCI: Lower and upper 95% confidence intervals

Model selection and contrasts for SCGx mice experiments (Figure 7)

Model selection on SCGx mice experiments									
Bottom up construction of models				anova between models (Test: Chisq)					
	Variables	AIC		Model	Resid df.	Resid. Dev	df	Deviance	Pr(>Chi)
Model 1	Contrast	107		Model 1	58	44.58			
Model 2	Contrast + Treatment	99		Model 2	57	34.40	1	10.18	0.0014

Supplementary table 10: Model selection for experiments on SCGx mice. Model 2 was chosen as the best model.

Contrasts between factors for the best model							
Fixed effect		Prob	Std. Error	LCI	UCI	Odds Ratio	p
Treatment	Sham	0.48	0.061	0.37	0.61	0.31	0.0021
	SCGx	0.23	0.049	0.15	0.34		

Supplementary table 11: Contrasts performed for model 2. LCI and UCI: Lower and upper 95% confidence intervals
